# Novel Image Encryption Scheme Based on Fireworks Algorithm and Reversible Convolution

**DOI:** 10.3390/e28090995

**Published:** 2026-09-06

**Authors:** Yaru Liang, Bo Peng, Renxin Liu, Huamao Zhou, Nanrun Zhou, Xingtong Wu

**Affiliations:** 1 School of Engineering, Jiangxi Agricultural University, Nanchang 330045, China; liangyaru@jxau.edu.cn (Y.L.); liurenxin1@163.com (R.L.); jxauzhm@jxau.edu.cn (H.Z.); 2 Jiangxi Engineering Research Center of Animal Husbandry Facility Technology Exploitation, Jiangxi Agricultural University, Nanchang 330045, China; 3 School of Software, Jiangxi Agricultural University, Nanchang 330045, China; 18942377255@163.com; 4 School of Electronic and Electrical Engineering, Shanghai University of Engineering Science, Shanghai 201620, China; nrzhou@sues.edu.cn

**Keywords:** fireworks algorithm, convolution operation, image encryption, information security

## Abstract

As information technology evolves rapidly, image data is exposed to growing risks of security breaches and privacy leaks during transmission and storage. Therefore, image encryption has attracted significant attention as an effective protection measure. Nevertheless, most chaos-driven image encryption schemes suffer from inferior chaotic randomness, making them prone to cryptanalytic cracking in practice. To solve this problem, a new image encryption scheme is proposed by integrating the fireworks algorithm with a convolution operation. First, the original image is permuted via the Arnold transform and an improved permutation strategy. Then, the classical Logistic map is iterated to generate an initial pseudo-random sequence, which is further optimized by the fireworks algorithm. Finally, a reversible convolution operation is integrated with a bit-level diffusion mechanism to achieve image encryption. Experimental results confirm that the proposed scheme exhibits superior performance in terms of statistical analysis, robustness analysis, and image-quality assessment, and it possesses remarkable security against various cryptanalytic attacks.

## 1. Introduction

With the continuous advancement of digitization, communication modes are no longer limited to traditional carriers such as letters and telephones. Images, owing to their rich information content and convenience in transmission and storage, have become an important medium for data interaction. However, image data usually contain sensitive information during network transmission and sharing, including personal privacy, transaction security, military confidential information, and national security [1]. If unauthorized users access, steal, or tamper with image data arbitrarily, severe consequences will ensue. Therefore, how to effectively guarantee the security and privacy of image data has become a current research hotspot. As an effective solution to the above problem, image encryption generally involves two steps: scrambling and diffusion. Scrambling restructures the spatial distribution of image pixels, while diffusion suppresses the correlation of adjacent pixels through pixel value adjustment to conceal the original image information [2]. Existing image encryption research mainly focuses on the generation of random chaotic sequences and the structural design of encryption algorithms [3]. Cryptography and chaos theory exhibit intrinsic structural similarities, which facilitates the application of chaos theory to image encryption, particularly for generating pseudo-random sequences. The application of chaotic systems in digital image encryption can be traced back to Fridrich’s work in 1998, where chaotic key streams were generated for pixel perturbation [4]. Since then, people have continuously explored the applications of chaos theory to image encryption. Alaklabi et al. used the Logistic map to generate random sequences for image scrambling and diffusion [5]. Meanwhile, they proposed an E-Chirp Z-Transform to tackle the limitation that the chaotic trajectories generated by the 1D Logistic map are susceptible to prediction. Unfortunately, this method inevitably leads to a decline in encryption efficiency. Furthermore, Tu et al. proposed an image encryption scheme by combining the 2D Logistic map with DNA sequences [6]. The pseudo-randomness of chaotic sequences can be enhanced by raising the dimension. Chaotic sequences combined with DNA sequences were introduced into the Feistel encryption architecture. Although the key space was sufficiently large, the encryption process was relatively complicated. To reduce this complexity, Yang et al. constructed an image compression and encryption scheme by virtue of integrating a hyper-chaotic system with BP neural networks, thereby improving the transmission efficiency [7]. However, the encryption algorithm mainly relies on the hyper-chaotic system to generate pseudo-random sequences, resulting in strong reliance on chaotic systems and stringent performance requirements for such chaotic systems. Wang et al. combined a logarithmic Logistic map with fractional Fourier transform to encrypt images [8]. This method broadens the search range, enhances the sensitivity of algorithm parameters, enriches the structure of the algorithm, and improves the security performance. Nevertheless, this algorithm suffers from high computational complexity. Ye et al. designed a multi-image encryption and hiding scheme using a novel 3D coupled chaotic system [9]. The scheme employs a 3D chaotic system and cross-plane operations for image encryption. Embedding multiple encrypted images inevitably changes the pixel values of carrier images, resulting in the degradation of image quality. In addition, there are many typical image encryption schemes based on compressed sensing [10,11,12,13,14], frequency domain transform [15,16,17,18,19,20], optics [21,22,23,24], cellular automata [25,26,27], and other methods with chaotic systems. Nonetheless, few studies have investigated image encryption algorithms that are constructed from chaotic systems and the fireworks algorithm. The fireworks algorithm (FWA) possesses excellent global optimization capability and is widely applied to tackle various optimization problems. FWA is mainly composed of four parts, i.e., the explosion operator, the variation operator, the mapping principles, and the selection strategy. Apart from the selection strategy, the other three components need random numbers for firework-explosion calculations. Therefore, the Euclidean distances among firework individuals, explosion sparks, and the variant sparks exhibit high randomness. Meanwhile, FWA features explosiveness, diversity, and fast convergence [28,29].

In this paper, an image encryption algorithm is developed by combining FWA with convolution operations. The pseudo-random sequence generated by the 1D Logistic map is divided into several parts and then fed into FWA to generate a new pseudo-random sequence. This approach not only inherits the high computational efficiency of low-dimensional chaotic systems, but also avoids the risk of secret key cracking. Furthermore, it effectively enhances the randomness of the generated sequence. In addition, a keypad-based image scrambling method is proposed, which imparts higher randomness and complexity to the encryption process and increases the difficulty of illegal cryptanalysis. Subsequently, one part of the generated pseudo-random sequence is used for target image diffusion in convolution operations, while the other part is applied to bit-level diffusion. Finally, experimental results demonstrate that the proposed algorithm achieves excellent performance in many aspects, such as grayscale histogram distribution, information entropy, encryption quality evaluation, and signal-to-noise ratio analysis. Thus, it exhibits overall satisfactory security and practicality.

## 2. Related Knowledge

### 2.1. Logistic Map

The classical 1D logistic map is defined as [30]:

(1) $$Q_{n+1}=\mu Q_{n}(1-Q_{n})$$

where $Q_{n}$ and $Q_{n+1}$ are the sequence values of chaos, and μ is the control factor ranging from 0 to 4. The system falls into a chaotic state when 3.5966≤μ<4. The range of the initial value $Q_{0}$ is (0, 1). Current low-dimensional chaos-based image encryption methods suffer from several drawbacks, including fixed parameter settings, insufficient key space, and low complexity [31]. To effectively enhance the security performance of the image encryption algorithms and optimize their encryption structure, the fireworks algorithm (FWA) is adopted in this paper. After configuring parameters including the number of fireworks, the number of variant sparks, and the radius of the explosion, the sparks generated by the explosion are regarded as new fireworks for subsequent iterative explosions. The Euclidean distances between explosion-generated sparks are applied as pseudo-random numbers. The pseudo-random numbers controlling FWA are generated by the 1D Logistic map, which compensates for its insufficient randomness.

### 2.2. Fireworks Algorithm

The fireworks algorithm (FWA) [32], proposed by Tan in 2010, is mainly applied to optimization problems. By initializing the number and positions of fireworks with different explosion radii, sparks at different distances are generated. This explosion mechanism randomly scatters sparks across the area surrounding the initial firework. Superior fireworks are allocated more search resources with a smaller explosion range, while inferior ones are assigned fewer resources within a larger explosion range. Hence, superior fireworks perform effective local search in their neighboring regions to seek the optimal solution [33]. FWA consists of three core components: the explosion operator for generating explosion sparks, the mutation operator for producing Gaussian mutation sparks, and the selection strategy for determining the next generation of fireworks.

(1) Explosion operator

Firstly, a certain number of fireworks are initialized, and the fitness value of each firework’s position is calculated. A smaller fitness value indicates better performance and generates more sparks after explosion, which facilitates local search. Conversely, fewer resources are obtained from the explosion, leading to ordinary local-search capability. The explosion radius $A_{i}$ and the number of sparks *S_i_* for each firework $x_{i}$ are calculated as

(2) $$A_{i}=\delta \cdot \frac{f(x_{i})-y_{\min}+\varepsilon }{\sum_{i=1}^{N}\left(f(x_{i})-y_{\min}+\varepsilon \right)}$$

(3) $$S_{i}=\tau \cdot \frac{f(x_{i})-y_{\max}+\varepsilon }{\sum_{i=1}^{N}f(x_{i})-y_{\max}+\varepsilon }$$

where $y_{\min}=\min(f(x_{i}))\;(i=1,\;2,\;\ldots ,\;N)$ denotes the minimum fitness value of the *N* current fireworks, $f(x_{i})$ denotes the function of the fitness value for the firework, and $y_{\max}=\max(f(x_{i}))\;(i=1,\;2,\;\ldots ,\;N)$ denotes the maximum fitness value of the *N* current fireworks. δ is a constant used to adjust the explosion radius, τ is a constant that regulates the number of sparks, and ε is a tiny constant preventing the denominator from being 0.

To avoid generating excessive sparks, the sparks boundary constraint denoted by $S_{i}$ is adopted:

(4) $$S_{i}=\left\{\begin{array}{l} \mathrm{round}(a\cdot \tau ),\;S_{i}<a\cdot \tau \\ \mathrm{round}(b\cdot \tau ),S_{i}>b\cdot \tau \;\;\;\;\;(a<b<1)\\ \mathrm{round}(S_{i}),\;\;\;\;another \end{array}\right.$$

where a and b are constants, and round denotes the rounding function.

(2) Mutation operators

The mutation operator generates mutation sparks by first selecting one firework $x_{i}$ from the population, and then choosing certain dimensions of the firework for mutation. For a firework $x_{i}$ of *k* dimensions, the mutation operation is performed as

(5) $$x_{\mathrm{ik}}^{1}={\;x}_{\mathrm{ik}}\;\times \;e$$

where *e* stands for a random value derived from the chaotic system.

To prevent the spark region from expanding outward during explosions, the range of sparks is constrained as follows:

(6) $$x_{ik}^{1}=x_{LB,k}+\left|x_{ik}^{1}\right|\%(x_{UB,k}-x_{LB,k})$$

where % denotes the complementation operation, and $x_{\mathrm{UB},k}$, $x_{\mathrm{LB},k}$ are the upper and lower bounds of the k-dimensional solution space, respectively.

(3) Selection strategy

After the fireworks explode, explosion sparks and mutated sparks are generated. Several sparks are selected as new fireworks for subsequent iterations, and these selected individuals form the set *K*. The Euclidean distance between each selected spark is written as

(7) $$R(x_{i})=\sum_{x_{j}\in K}d(x_{i}-x_{j})=\sum_{x_{j}\in K}\left\|x_{i}-x_{j}\right\|$$

where *R*($x_{i}$) represents the sum of distances from the selected firework $x_{i}$ to all remaining individuals in the set *K*. When sparks are densely clustered around a given spark, its selection probability decreases. The corresponding probability is

(8) $$p(x_{i})=\frac{R(x_{i})}{\sum_{x_{j}\in K}x_{j}}$$

### 2.3. Fireworks Algorithm Controlled by the Logistic Map

For the given chaotic parameters, the 1D Logistic map is iterated to generate a random sequence. The previous 5000 entries are discarded to obtain the sequence $N_{1}$. Divide $N_{1}$ into six sequences, namely $n_{1},\;n_{2},\;n_{3},\;n_{4},\;n_{5}$ and $n_{6}$, which are then taken as the input of FWA. The computational procedure of FWA is shown in Algorithm 1. The main parameters of FWA include the initial number of fireworks *N*, the dimension *D*, the fitness value $\;f\left(x_{i}\right)$, the explosion radius $A_{i}$, the number of explosion-generated sparks $S_{i}$, the upper boundary $x_{\mathrm{UB}}$ and the lower boundary $x_{\mathrm{LB}}$ of the spark explosion location.
**Algorithm 1.** Partial fireworks algorithm: generating pseudo-random numbersInputs: $n_{1},\;n_{2},\;n_{3},\;n_{4},\;n_{5},\;n_{6}$. Output: the sequence of pseudo-random numbers $N_{2}$. Start:  1. Choose the series $n_{1}$ and initialize the positions of the *N* fireworks $x_{i}$;  2. For *i* = 1 to *N,*  3. Calculate the fitness value of each firework $f(x_{i})$, explosion radius $A_{i}$, and the number of sparks $S_{i}$ according to Equations (2) and (3);  4. End. // Generate sparks through explosion operations.  5.  For *i* = 1 to *N*,  6.       For j = 1 to $S_{i}$,  7. Store sparks; $x_{i}^{1}\;{=\;x}_{i}$;  8. Choose z dimensions as the set ${\mathrm{DS}}_{1},\;z\;=\;\mathrm{round}(D\;\times \;U)$, D denotes the dimension of $x_{i}$ and *U* is replaced by pseudo-random numbers in $n_{2}$ normalized from 0 to 1;  9. Calculate the position offset $h=\;A_{i}$ × *U*, where *U* is replaced by a pseudo-random number in $n_{3}$ normalized to [−1, 1];  10.     For each *k* in *DS*_1_,  11.       $x_{\mathrm{ik}}^{1}\;={\;x}_{\mathrm{ik}}\;+\;h$;  12.       According to Equation (6), map out-of-bounds sparks to the valid boundary range.  13.     End  14.  Preserve $x_{\mathrm{ik}}^{1}$ in exploding spark populations;  15.  End  16. End // produce $M^{1}$ mutated sparks $n_{4}$;  17.   For $i=\;1\;\mathrm{to}\;M^{1}$ do  18. Select a spark $x_{i}$ randomly;  19. Randomly choose *z* dimensions as the set ${\mathrm{DS}}_{2},\;\mathrm{where}\;z\;=\;\mathrm{round}(D\;\times \;U)$, D is the dimension of $x_{i}$, and *U* is replaced by a pseudo-random number from $n_{5}$ normalized between 0 and 1. Calculate the mutation parameter *e*, where *e* is the pseudo-random number derived from $n_{6}$;  20.    For each *k* in *DS*_2_, do  21.     ${\;x}_{\mathrm{ik}}^{2}\;={\;x}_{\mathrm{ik}}\;\times \;e$;  22.    Detect the boundary according to Equation (6) and map by the given rule;  23.    End  24. Save $x_{\mathrm{ik}}^{2}$ to the mutated spark population;  25. End  26. Preserve the Euclidean distances between any pair of fireworks, sparks, and mutated sparks, denoted by $N_{2}$;  27. Select individuals among fireworks, sparks, and mutated sparks as the next explosion fireworks according to Equation (8);  28. End

### 2.4. Performance Analysis of Random Sequences Generated by the FWA

#### 2.4.1. Sequence Distribution

In the fireworks algorithm (FWA), the generated sequence consists of the Euclidean distances between every pair of initial fireworks, explosion-produced sparks, and sparks from the mutated spark explosion. Therefore, FWA employs the pseudo-random sequence generated by the Logistic map to initialize the positions, dimensions, and positional offsets of the fireworks. Each module of FWA exhibits strong randomness in every iteration. Furthermore, the introduction of multiple parameters such as explosion boundaries enables the image encryption based on FWA to better resist nonlinear prediction attacks. The sequence $N_{2}$ generated by FWA is normalized according to Equation (9).

(9) $$N_{2}=\mathrm{floor}\left(\mathrm{mod}\left(N_{2}\;\times {\;10}^{8},\;256\right)\right)$$

where floor represents rounding down toward 0 and mod denotes the modulo operation that normalizes the sequence to the range [0, 255].

**Figure 1:**
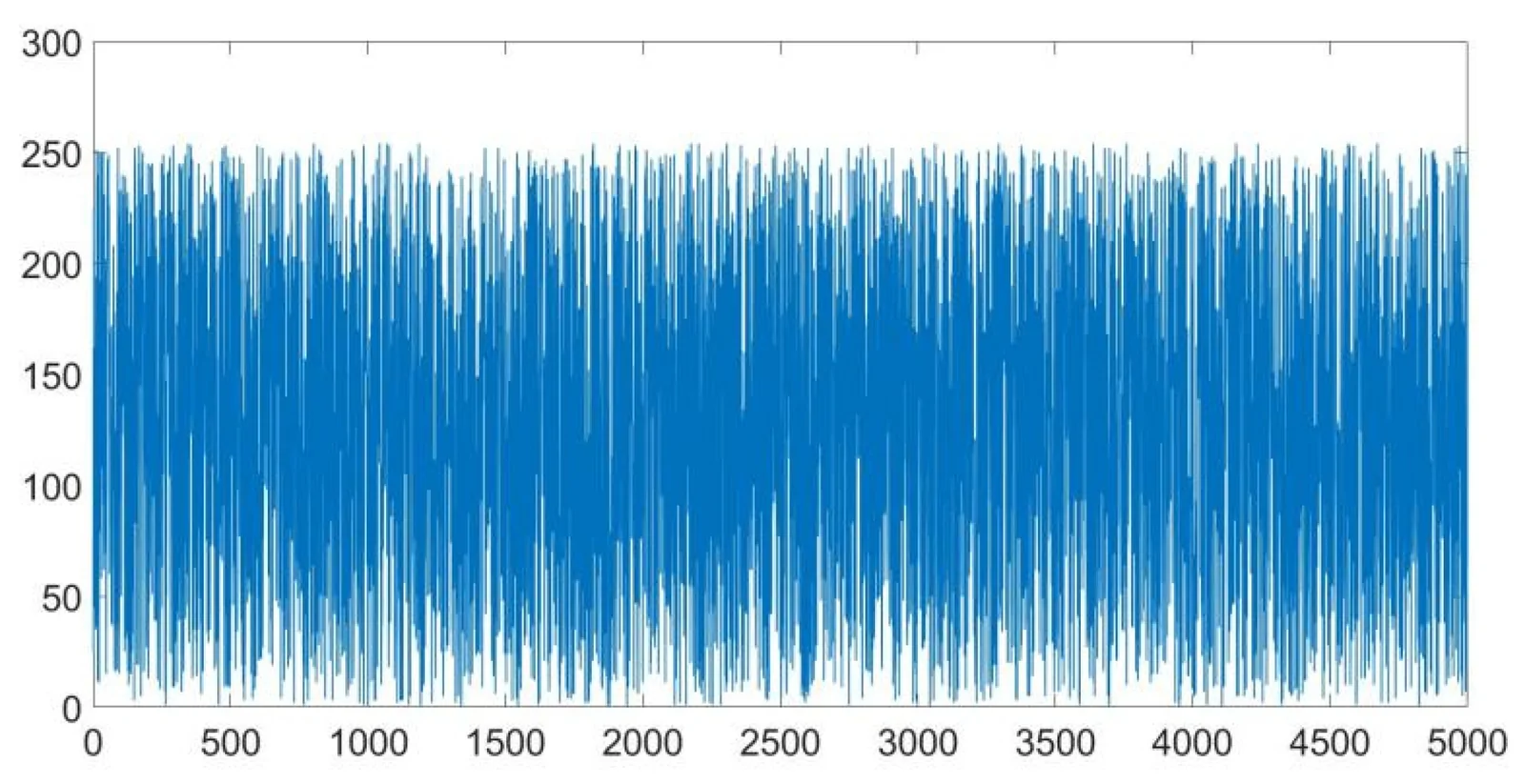
Output sequence plot of FWA.

5000 consecutive entries are selected from the generated pseudo-random sequence according to Equation (9), as displayed in Figure 1. It can be seen that the sequence is relatively uniformly distributed from 0 to 255.

#### 2.4.2. NIST Test

This section employs the NIST SP800-22 [8] randomness test suite to evaluate the performance of the proposed pseudo-random sequence. A one-million-bit sequence is tested with a significance threshold of 0.01. A test is considered valid if its *p*-value exceeds this threshold. In Table 1, √ indicates passing the NIST test, while × indicates failure to pass the NIST test. As shown in Table 1, all test items are qualified, which verifies that the generated sequence meets the randomness-based security requirements.

## 3. Encryption and Decryption Scheme

### 3.1. Encryption Process

**Figure 2:**
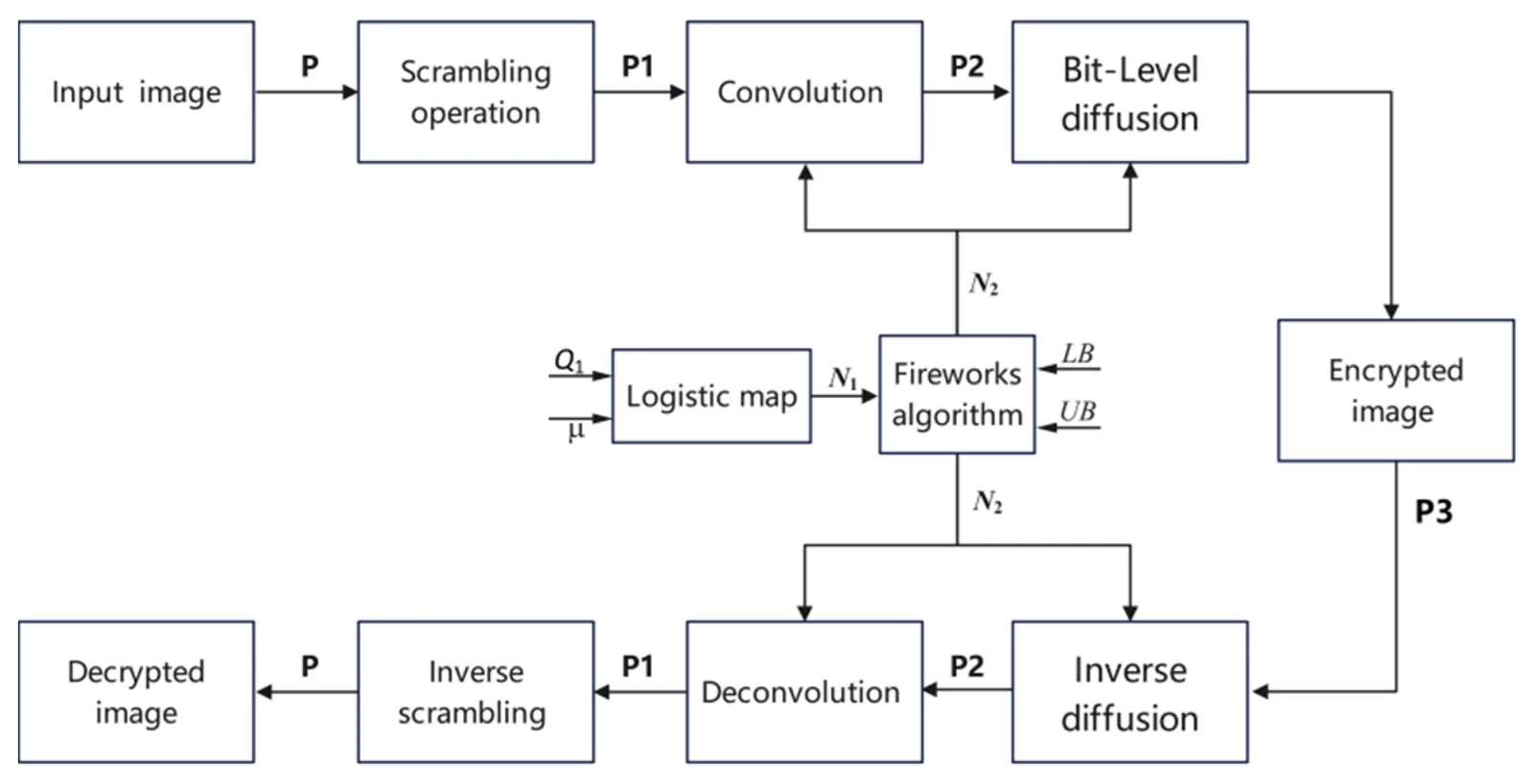
Diagram of the encryption process.

The encryption process fundamentally consists of two procedures, i.e., scrambling and diffusion. During the permutation process, the Arnold transform and a novel scrambling method are adopted to disturb the pixel positions of the image. Subsequently, FWA driven by a chaotic system is employed to generate pseudo-random numbers, which are then applied to convolution operations to achieve diffusion. The overall block diagram of encryption is shown in Figure 2.

The detailed encryption process is described below.

(1) Scrambling operation

**Figure 3:**
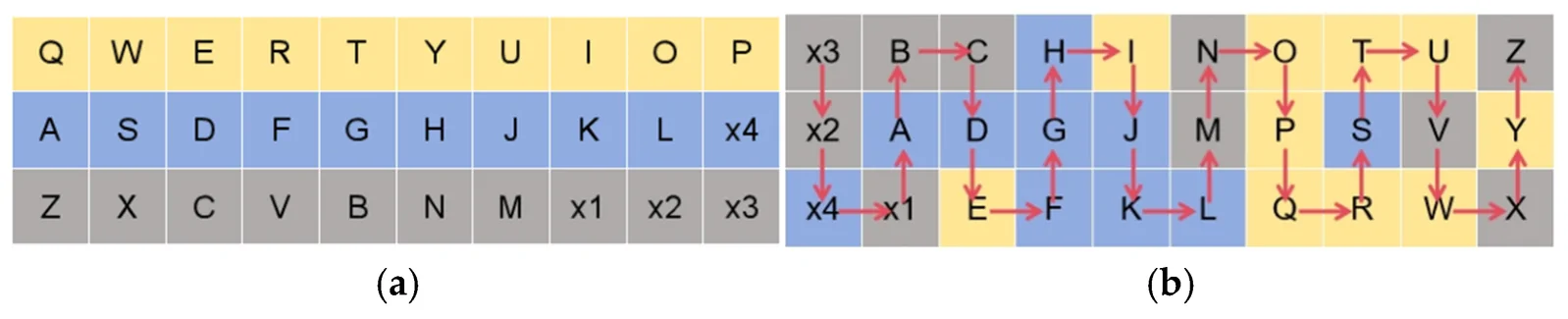
Scrambling operation rule: (**a**) the original location, (**b**) the scrambled location.

Since the Arnold transform is periodic, the scrambled image can be recovered after a specific number of iterations [34]. In order to eliminate the periodicity of the Arnold transform, the novel scrambling operations are performed to obtain P1. Assuming the standard matrix size is 3 × 10, the shuffled matrix is rearranged to match this standard size. The process of scrambling is exhibited in Figure 3.

(2) Generating pseudo-random numbers

Step 1: Select the parameter μ of the chaotic system. After iterating via Equation (1), a sequence of m + n terms is generated, and the first m terms are discarded to acquire the random sequence $N_{1}$. Subsequently, $N_{1}$ is divided into six parts, i.e., $n_{1},\;n_{2},\;n_{3},\;n_{4},\;n_{5}$ and $n_{6}$, with a total number of n elements.

Step 2: Set the parameters of FWA: the number of initial fireworks *m*_1_, variable dimension *D*, the number of variant sparks $M^{1}$, the number of explosions $S_{i}$, explosion radius $A_{i}$, the explosion constraint factors *a* and *b*, the number of iterations number *T*, the upper boundary *UB,* and the lower boundary *LB* of the spark explosion location. The sequence $N_{2}$ is generated according to Algorithm 1.

(3) Convolution stage

Multiple convolution operations are performed in the convolution phase. Taking two consecutive convolutions as an example, any column Lh with Lh≤M is selected from the scrambling matrix **P1** of size M × N. The convolution kernel size is *C* × *C*. To maintain the size of the output matrix unchanged after convolution, the target matrix needs to be zero-padded. In this example, zeros are padded at the bottom of the matrix during calculation. A set of pseudo-random numbers generated by FWA is selected to form the matrix M1 of size (M+C−1)×N.

Table 2 lists the convolution operations of the same input matrix with different convolution kernels, including the scheme proposed in Ref. [35]. The convolution calculation in Ref. [35] can change pixel values, but does not alter the difference between adjacent pixel values. Therefore, it faces the risk of being cracked by attackers. Inspired by this approach, we propose an improved reversible convolution strategy. The detailed convolution steps are listed in Table 3. Through analysis, we conclude that the differences between adjacent pixel values before and after the convolution are irrelevant and exhibit no obvious regularity. Moreover, without the pseudo-random matrix **M1**, the convolved data cannot be recovered.

(4) Diffusion stage

The cipher matrix **P2** is obtained after multiple convolutions. An arbitrary column of the input matrix is fixed and excluded from convolution operations so that its information can be preserved. To further enhance encryption performance and eliminate residual correlation information, bit-level diffusion is introduced.

Step 1: Convert the element *p*_2i_ in **P2** (*i* = 1, 2, …, *M × N*) into an 8-bit binary string a_1_a_2_a_3_a_4_a_5_a_6_a_7_a_8_.

Step 2: Take several pseudo-random numbers from $N_{2}$ to construct a matrix **K1** of size M × N, which serves as the diffusion matrix for the higher four bits. Then, select several pseudo-random numbers from $N_{2}$ to form a matrix **K2** of size M × N as the diffusion matrix for the lower four bits, and convert all the elements in **K1** and **K2** into 8-bit binary numbers. Each element ${K1}_{i}$(i = 1, 2, …, M × N) is transformed into an 8-bit binary number, denoted by x_1_x_2_x_3_x_4_x_5_x_6_x_7_x_8_. Similarly, the binary form of ${K2}_{i}$(i = 1, 2, …, M × N) is expressed as y_1_y_2_y_3_y_4_y_5_y_6_y_7_y_8_.

**Figure 4:**
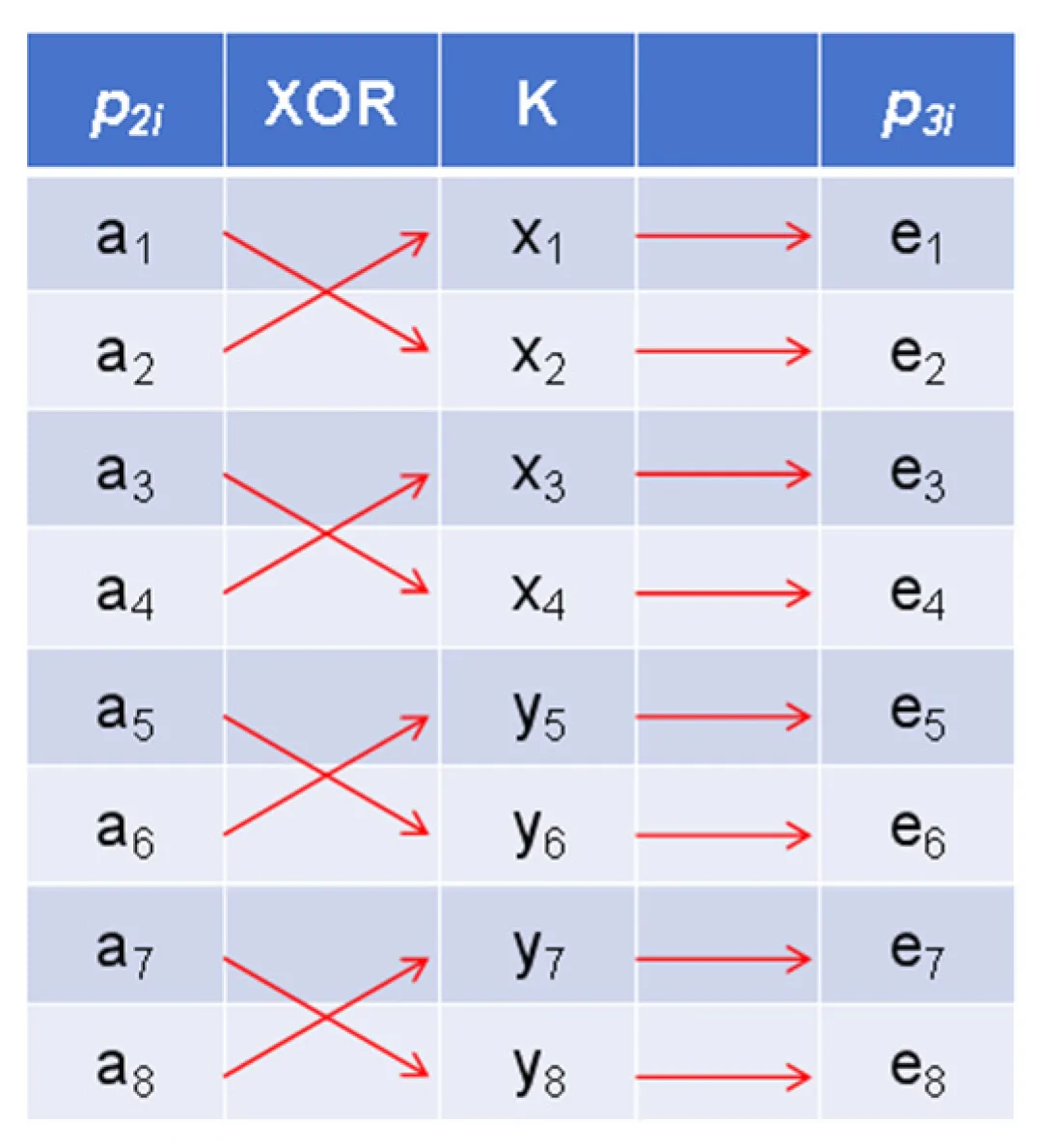
Bit-level diffusion.

Step 3: The high four bits (a_1_a_2_a_3_a_4_) of each element *p*_2i_ in **P2** correspond to x_1_x_2_x_3_x_4_ from the corresponding element in **K1**, whereas the low four bits (a_5_a_6_a_7_a_8_) correspond to y_5_y_6_y_7_y_8_ from **K2**. The diffusion operation is illustrated in Figure 4.

Step 4: After bit-level diffusion, an 8-bit binary number $e_{1}e_{2}e_{3}e_{4}e_{5}e_{6}e_{7}e_{8}$ is obtained. Subsequently, the binary values after bit-level diffusion are converted into decimal form to obtain the encrypted image **P3**.

### 3.2. Decryption Process

Due to the symmetry of the proposed encryption-decryption model, the decrypted image can be obtained by reversing the encryption procedure. An example of the deconvolution operation is shown in Table 4.

## 4. Experimental Results and Performance Analysis

### 4.1. Experimental Results

**Figure 5:**
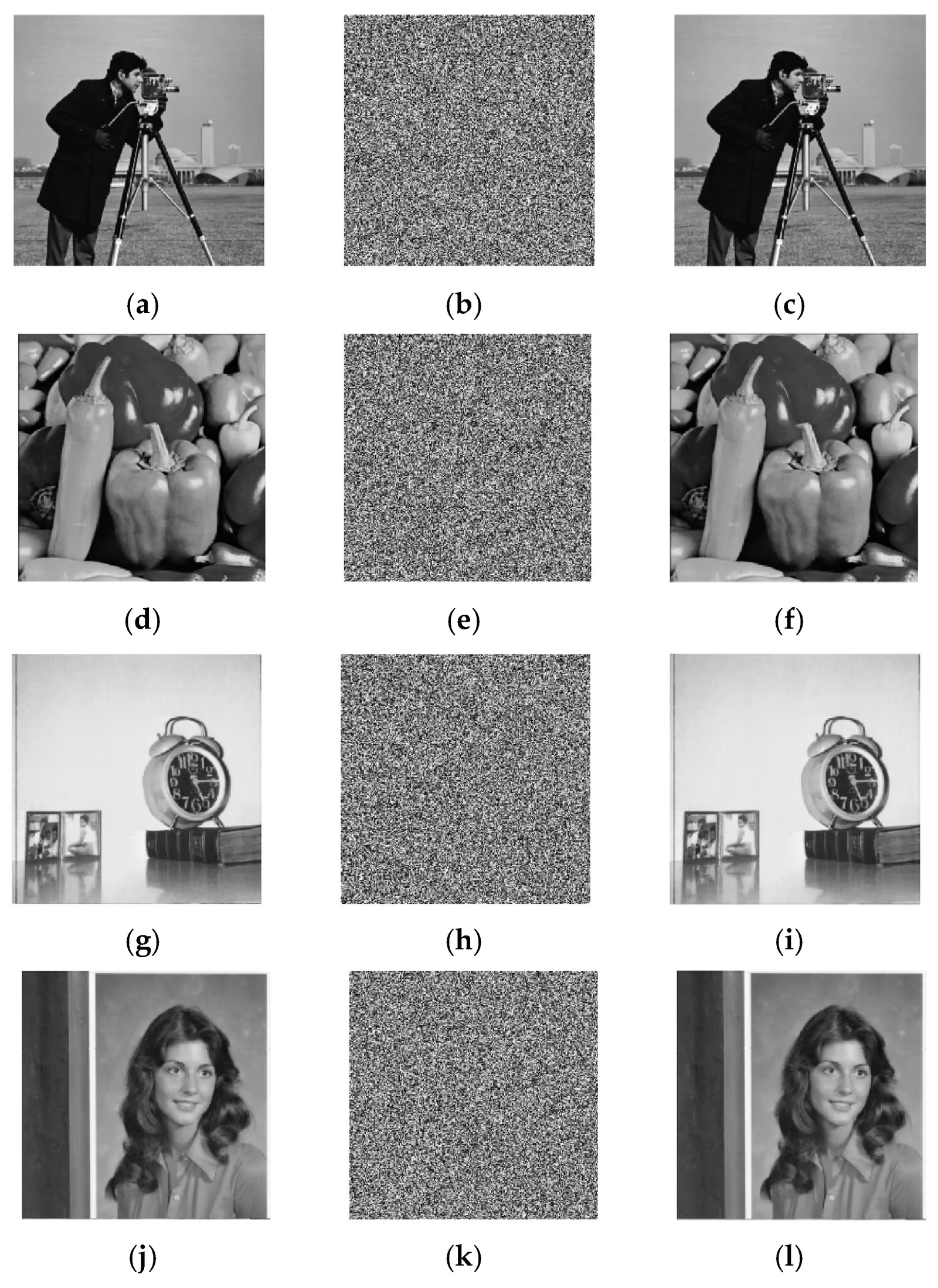

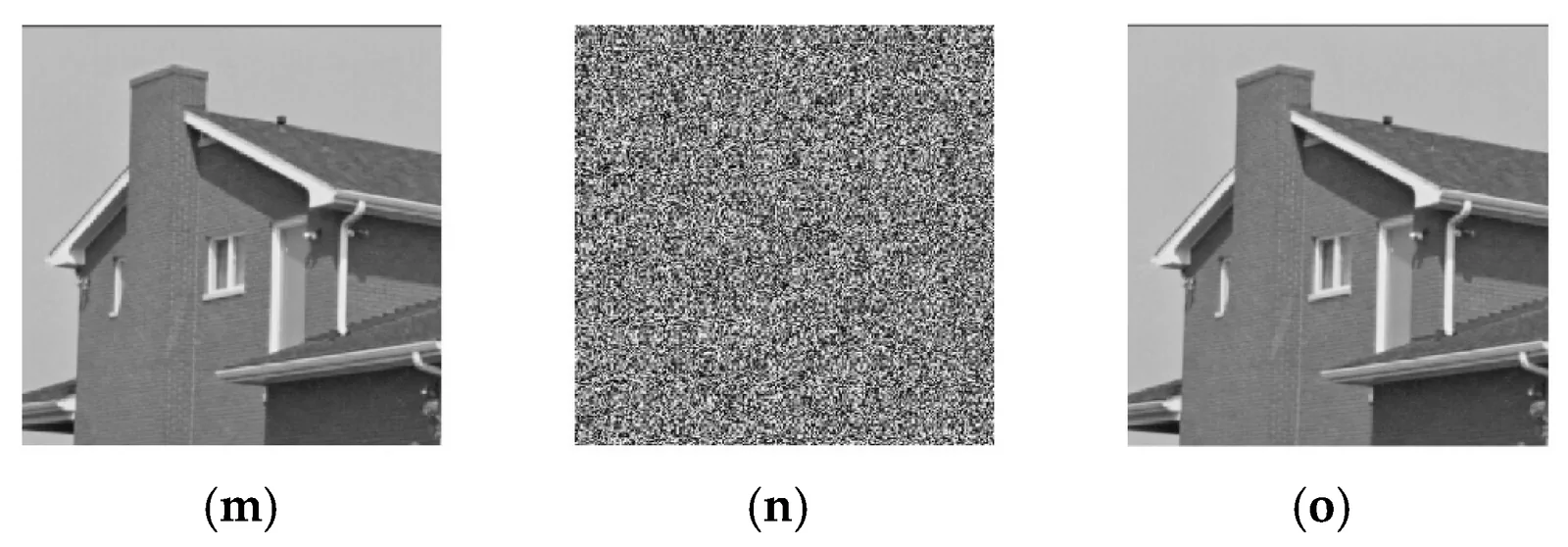
Images (**a**–**c**), (**d**–**f**), (**g**–**i**), (**j**–**l**), and (**m**–**o**) are the original, encrypted, and decrypted Cameraman, Peppers, Clock, Female, and House, respectively.

To evaluate the encryption performance, five standard grayscale test images, Cameraman, Peppers, Clock, Female, and House, all of size 256 × 256, are chosen from the public USC-SIPI standard image database. With diverse features and typical structural characteristics, they facilitate multi-dimensional assessment of the proposed encryption algorithm. All experiments were implemented in MATLAB 2022b on a 64-bit Windows 11 platform with 16 GB RAM. The keys are set as μ = 3.7245, $Q_{0}$ = 0.1, $x_{\mathrm{LB}}=$ [−10, −10], $x_{\mathrm{UB}}$ = [10,10], $\;{\mathrm{Lh}}_{1}$ = 25, ${\mathrm{Lh}}_{2}$ = 50, ${\mathrm{Lh}}_{3}$ = 125, and three different convolution kernels. Figure 5 illustrates the experimental results concerning encryption and decryption.

### 4.2. Histogram Analysis

**Figure 6:**
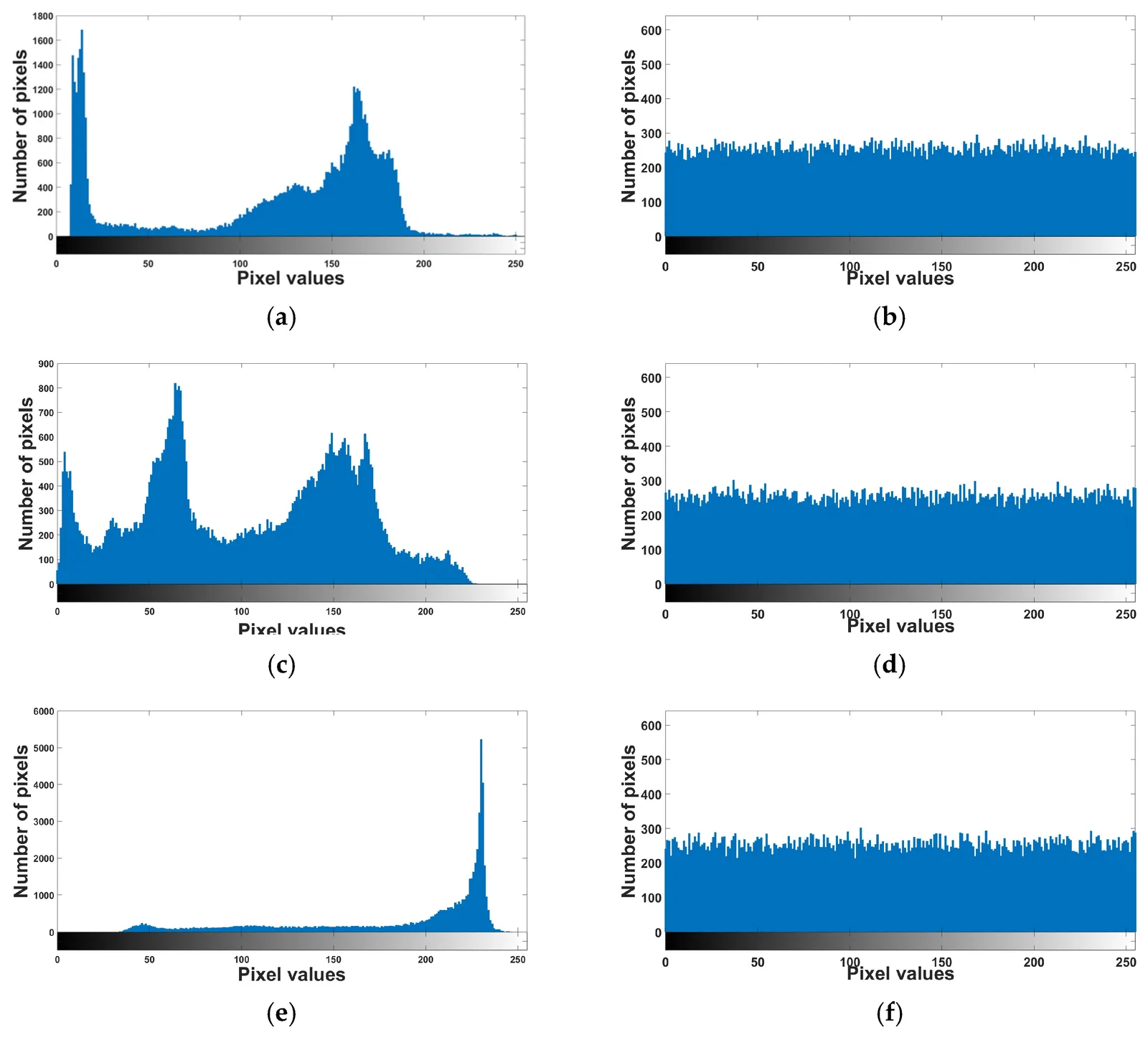

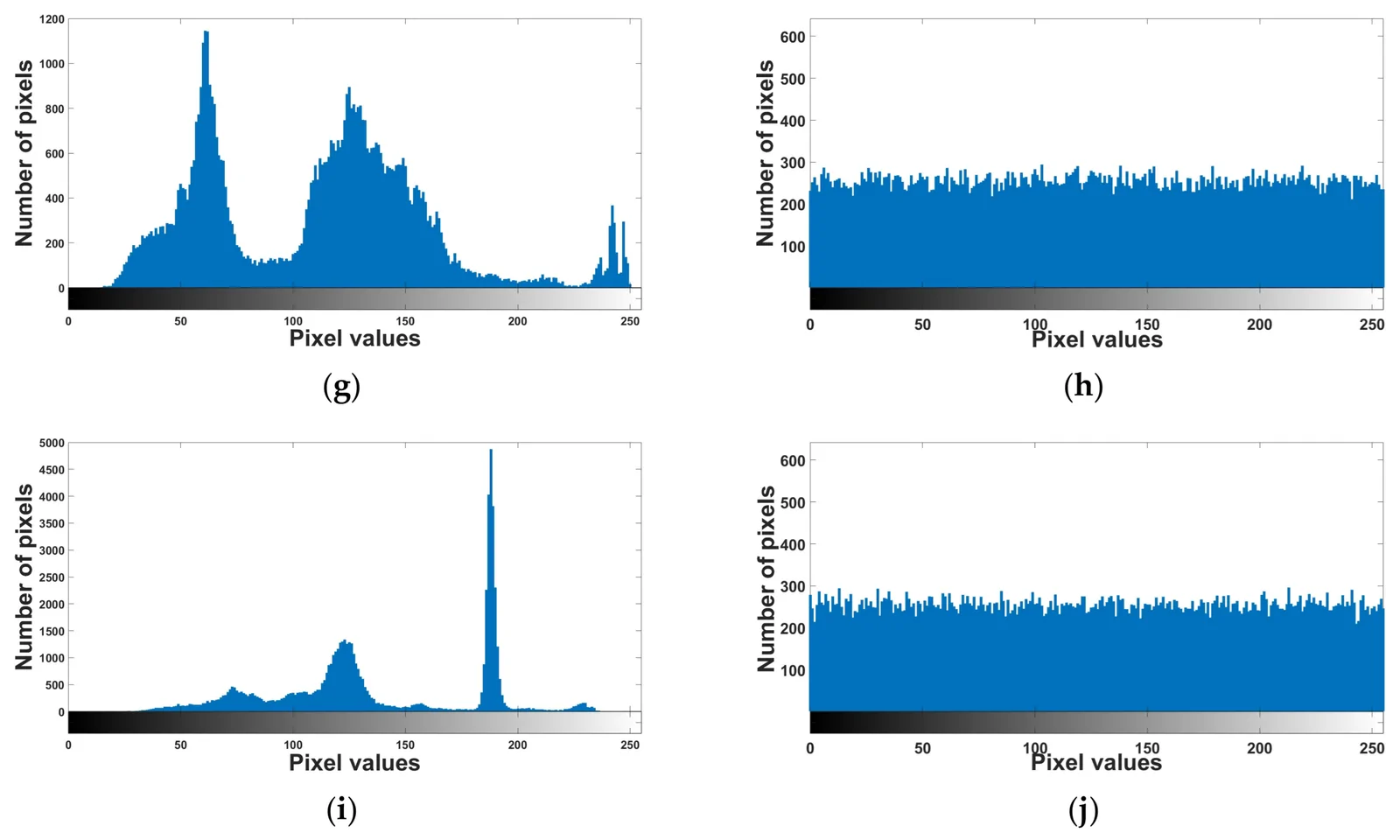
Images (**a**,**b**) are the histograms of the original and encrypted Cameraman, respectively. Similarly, images (**c**–**j**) correspond to the histograms of the original and encrypted Pepper, Clock, Female, and House, respectively.

Histograms provide a visual representation of pixel intensity distributions in images. An uneven distribution may allow attackers to extract effective information via statistical analysis [36]. The closer the occurrence probabilities of different pixel values are, the lower the risk of illegal information cracking is. Figure 6 plots the grayscale histograms of test images before and after two rounds of diffusion. A significant difference can be observed between the plaintext and ciphertext histograms. The former varies drastically, while the latter is relatively flat. Therefore, the encrypted images are difficult to crack or restore via statistical analysis.

### 4.3. Key Analysis

**Figure 7:**
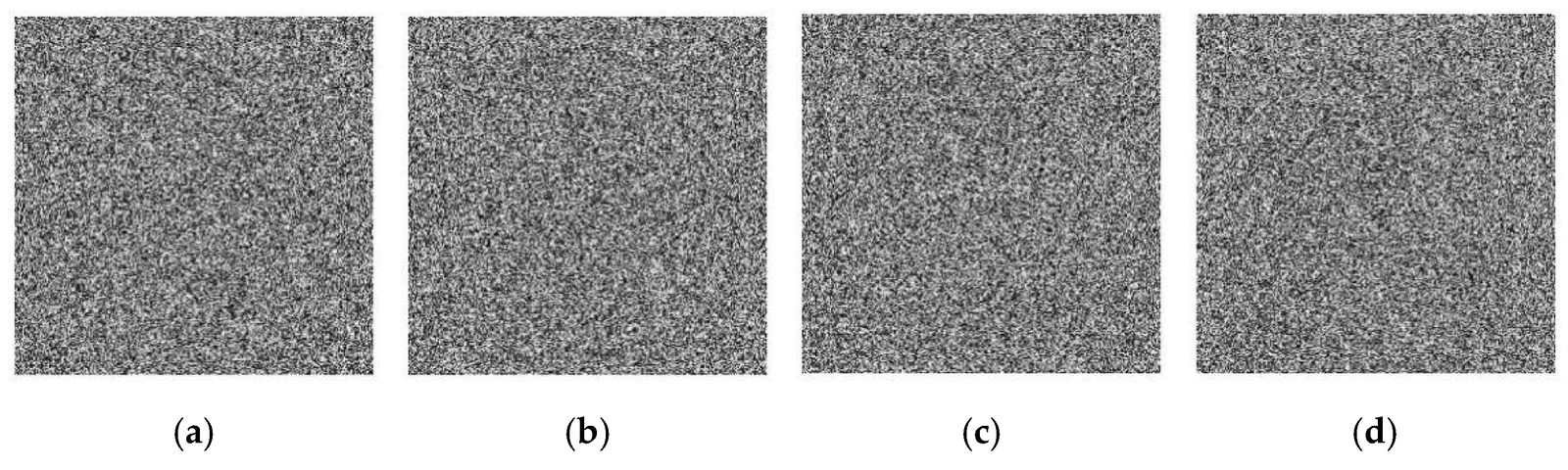
Decrypted images using an incorrect key. (**a**) $∆Q_{0}\;={\;10}^{-15}$, (**b**) $∆\mu \;={\;10}^{-15}$, (**c**) $∆\mathrm{LB}\;={\;10}^{-7}$, and (**d**) $∆\mathrm{UB}\;=\;10^{-7}$.

To ensure that a system can resist attacks, the magnitude of the key space also needs to meet certain requirements [37]. In the proposed method, both the chaotic initial values and chaotic parameters have a precision of 10^−15^, and the four critical values related to FWA boundaries have a precision of $10^{-7}$. Therefore, the computed key space is $10^{58}\approx 2^{192}$. Furthermore, the 21 parameters of the 3 × 3 convolution kernels are arbitrary positive integers, yielding a key space far larger than $2^{128}$. Consequently, the key space satisfies the security requirements and can effectively resist exhaustive attacks. The decrypted images obtained with a slightly deviated key are exhibited in Figure 7. As can be seen from the figure, the decrypted image remains chaotic, and no information is leaked, verifying that the proposed encryption algorithm is highly sensitive to tiny variations in the key.

### 4.4. Information Entropy Analysis

Information entropy is commonly used to evaluate the irregularity, unpredictability, and pixel intensity randomness of encrypted images, making it an important metric for assessing encryption performance. A higher degree of pixel distribution randomness corresponds to a greater information entropy value [38]. It can be calculated mathematically as

(10) $$H(s)=-\sum_{s}p(s_{i})\times \log_{2}p(s_{i})$$

where $p(s_{i})$ represents the occurrence probability of different pixel values $s_{i}$. A grayscale image with 256 gray levels corresponds to the optimal entropy *H*(*s*) of 8 bits. Table 5 lists the entropy results of different encryption algorithms. It can be seen that all results are extremely close to 8 bits. This indicates that the pixel distribution of the image is effectively scrambled after encryption, which further verifies that the proposed algorithm has excellent encryption performance and strong resistance to entropy-based attacks.

### 4.5. Adjacent Pixels Correlation

Attackers can illegally acquire plaintext content by analyzing the correlative features of adjacent pixels across horizontal, vertical, and diagonal directions, and thereby steal the plaintext information. To defend against such statistical attacks, the designed encryption algorithm needs to eliminate pixel correlations in all three directions [42]. To verify this, 5000 pairs of adjacent pixels are arbitrarily chosen from the Cameraman and Peppers test images. The correlation coefficient $r_{xy}$ is calculated via Equation (11) [43], and the results are presented in Table 6.

(11) $$r_{xy}=\frac{\sum_{i=1}^{U}\left(x_{i}-\bar{x})(y_{i}-\bar{y}\right)}{\sqrt{\sum_{i=1}^{W}(x_{i}-\bar{x}{)}^{2}\sqrt{\sum_{i=1}^{W}(y_{i}-\bar{y}{)}^{2}}}}$$

where U denotes the total number of randomly selected pixel pairs, while $\bar{x}$ and $\bar{y}$ stand for the mean of the x and y sequences, respectively. When the correlation coefficient of adjacent pixels is close to 0, the encrypted image demonstrates better randomness.

**Figure 8:**
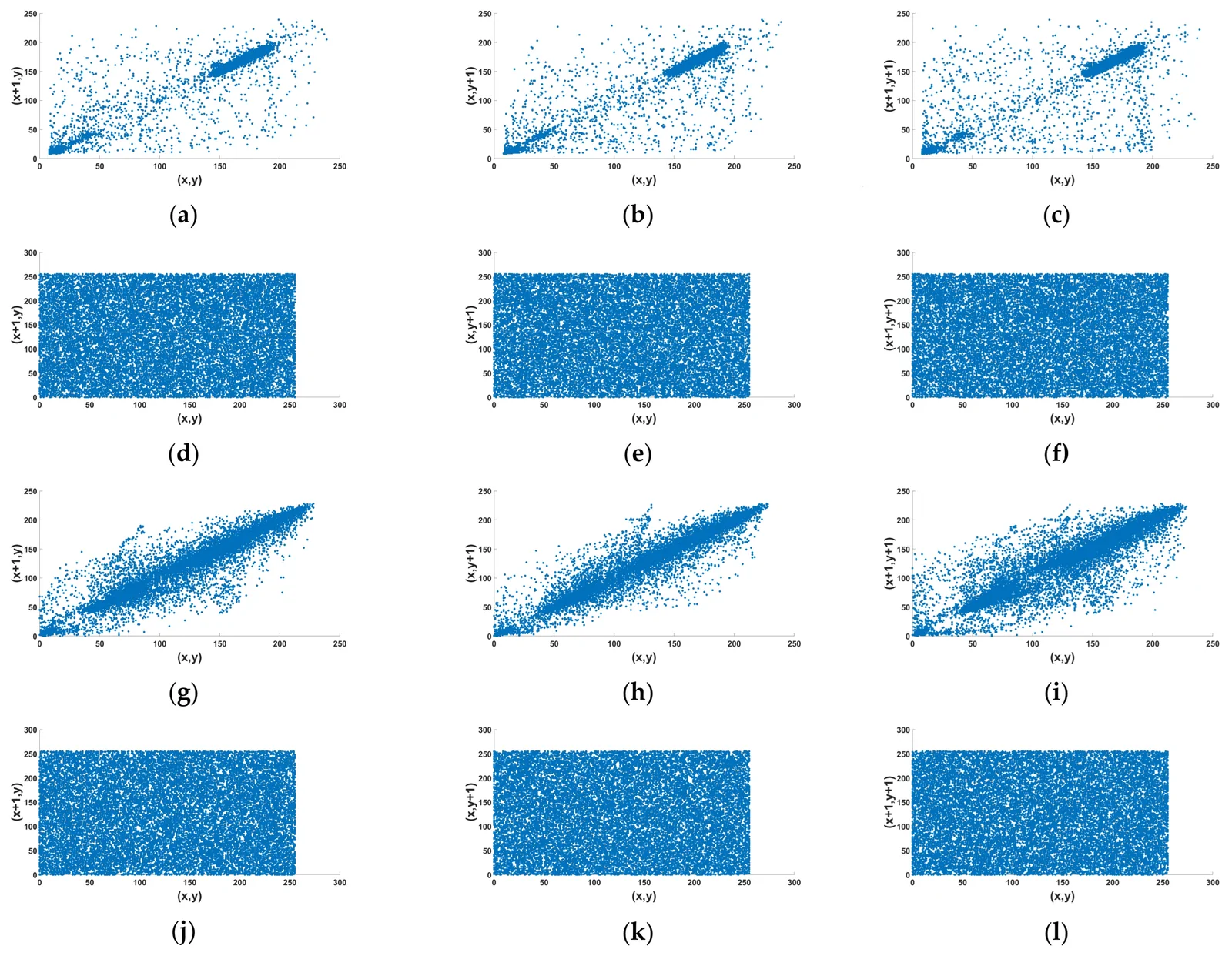
(**a**–**c**) show the correlation coefficients of the plaintext Cameraman in three different directions. (**d**–**f**) show the correlation coefficients of the encrypted Cameraman in three different directions. (**g**–**i**) show the correlation coefficients of the plaintext Peppers in three different directions. (**j**–**l**) show the correlation coefficients of the encrypted Peppers in three different directions.

It is evident that after several rounds of pixel scrambling, the correlation coefficients and scatter plots reveal a dramatic drop in adjacent pixel correlation for the test images. Furthermore, the correlation results of Cameraman are compared with those from other existing algorithms, demonstrating that the proposed algorithm achieves comparable performance. The correlation coefficients for all test images are reduced from nearly 1 to 0.001. The algorithm performs well in eliminating pixel correlations and distributing image pixels in a highly disordered manner, which enables it to effectively defend against statistical attacks from this perspective (Figure 8).

### 4.6. Analysis of Differential Statistics

Attackers usually introduce minor tampering to the original image. The tampered image is encrypted using the same key and encryption scheme. By comparing the two ciphertexts, attackers are able to extract valuable confidential information [44]. NPCR and UACI are two performance indicators for evaluating resistance against differential attacks. Their calculation equations are shown in Equations (12) and (14), respectively [45].

(12) $$\mathrm{NPCR}=\frac{\sum_{i,j}D(i,j)}{W\times H}$$

(13) $$D(i,j)=\left\{\begin{array}{c} 0\;\;if\;E(i,j)=E^{\prime }(i,j)\\ 1\;\;if\;E(i,j)\neq E^{\prime }(i,j) \end{array}\right.$$

(14) $$\mathrm{UACI}=\frac{\sum_{i,j}E(i,j)-\sum_{i,j}E^{\prime }(i,j)}{255\times W\times H}$$

where *W* and *H* denote the height and width of the image. D(i, j) and E(i, j) represent the pixel values of the original and the encrypted images, respectively. $\;E^{\prime }\left(i,\;j\right)$ represents the pixel value of the encryption result with one pixel modified in the plaintext. It is widely recognized as optimal when the NPCR value is between 99.3082 and 99.5906, and the UACI value is between 33.3115 and 33.6156 [46]. According to Table 7, all test images exhibit the NPCR and UACI values within the permissible ranges. After multiple rounds of global convolution operations, even a tiny change to one pixel in the plaintext will yield an encrypted image that is almost completely different from the original image. Hence, this verifies that the proposed encryption scheme possesses high complexity and excellent diversity.

### 4.7. Robustness Analysis

Encrypted images are vulnerable to malicious tampering and cropping during transmission. Hence, robustness analysis is of great significance. In this section, we calculate and evaluate the mean square error (MSE), peak signal-to-noise ratio (PSNR), and structural similarity index measure (SSIM) for robustness assessment.

#### 4.7.1. Cropping Attack Analysis

**Figure 9:**
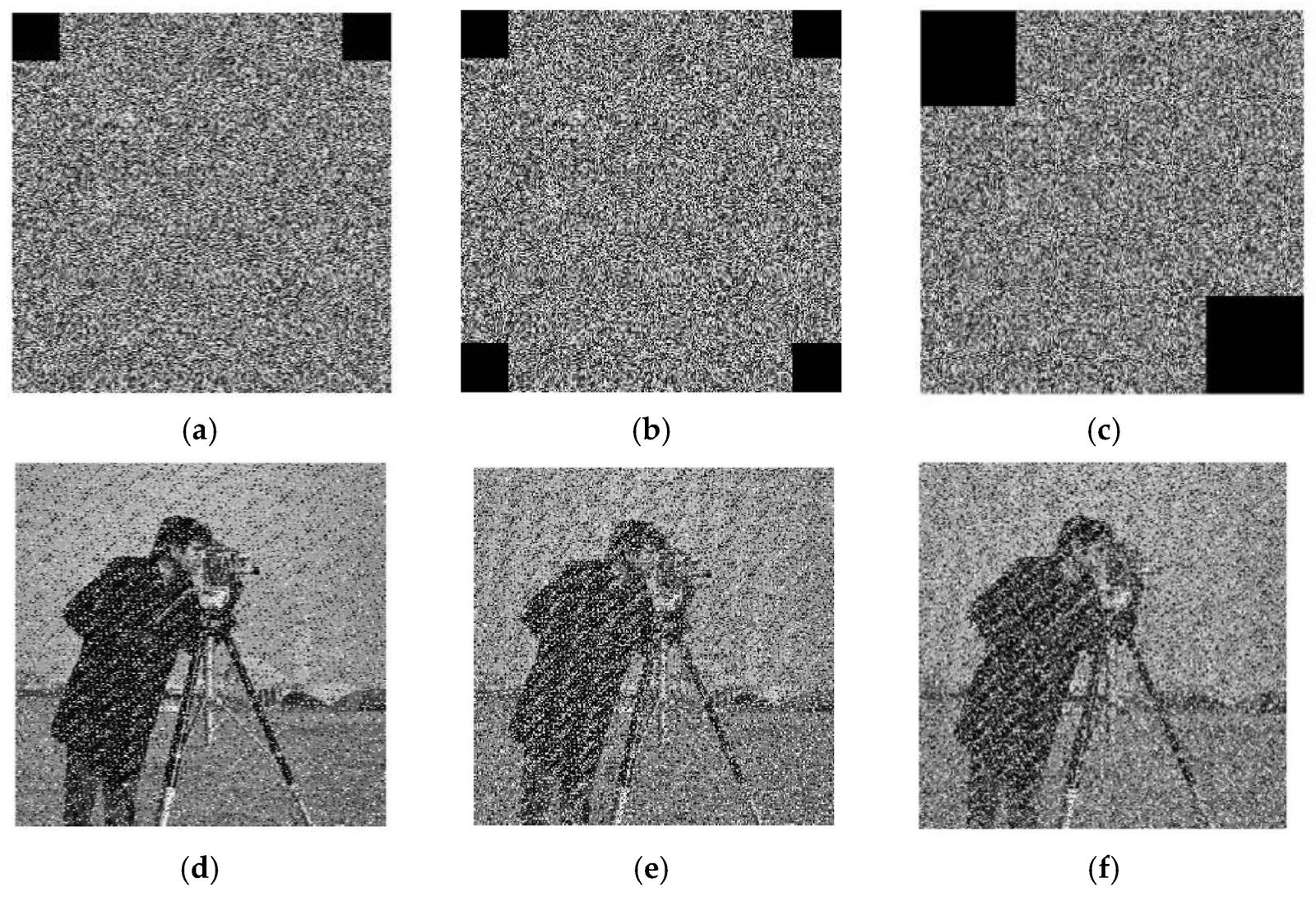
(**a**–**c**) correspond to cropping 1/32, 1/16, and 1/8 of the encrypted images, respectively, while (**d**–**f**) are the corresponding decrypted images.

During the transmission and storage of ciphertext images, partial information may be lost, so it is necessary to conduct a robustness test on the encryption algorithm. A practical encryption algorithm should not only achieve effective image encryption, but also maintain satisfactory decryption and reconstruction performance under partial information loss. To evaluate the algorithm’s resistance to cropping attacks, encrypted images are cropped into different sizes at various positions. The cropped encrypted images and their corresponding decrypted results are presented in Figure 9. Valid information can still be distinguished even under blurring interference. Therefore, the proposed scheme can still preserve valid image information against cropping attacks.

#### 4.7.2. Noise Attack Analysis

**Figure 10:**
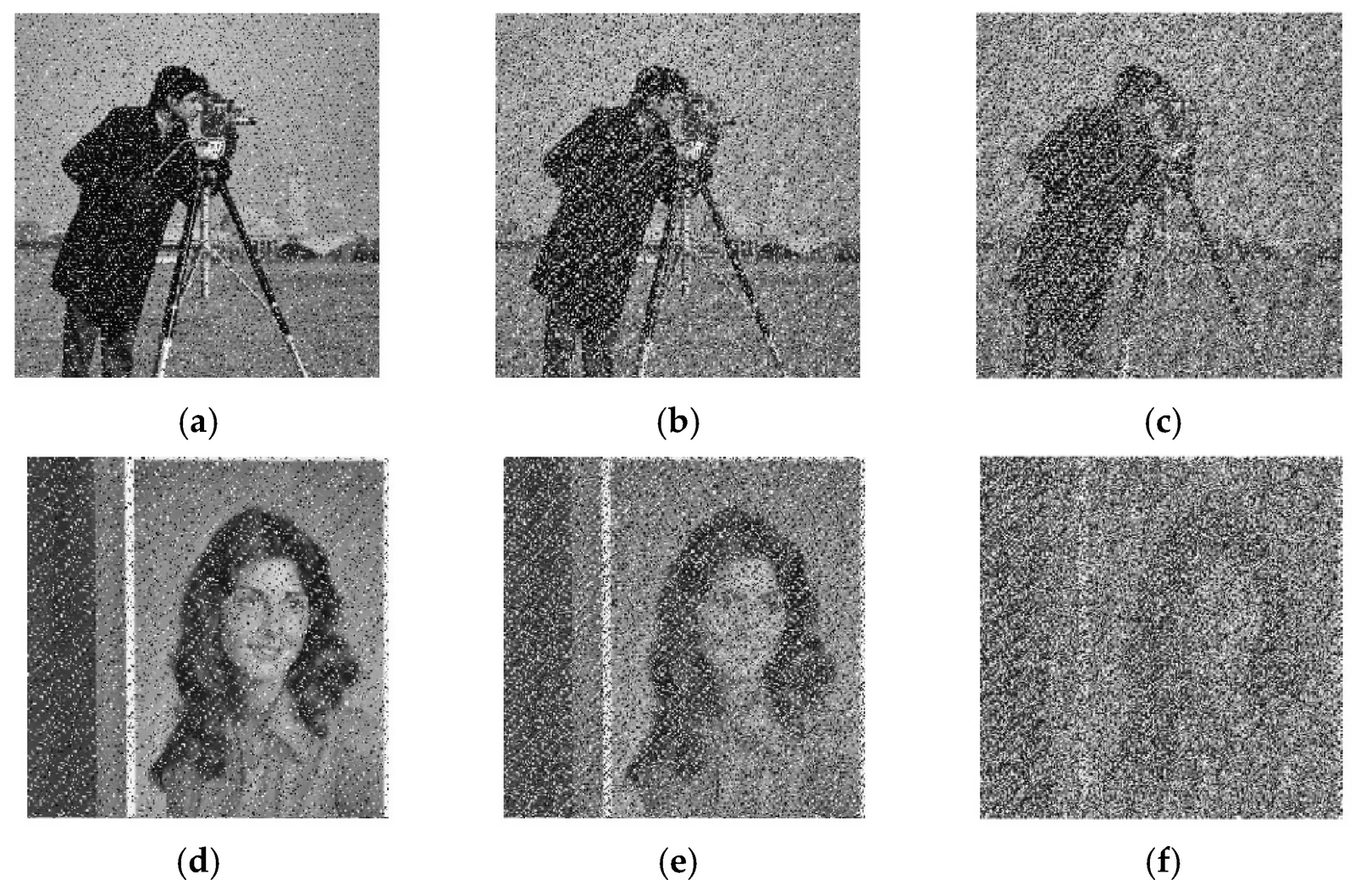

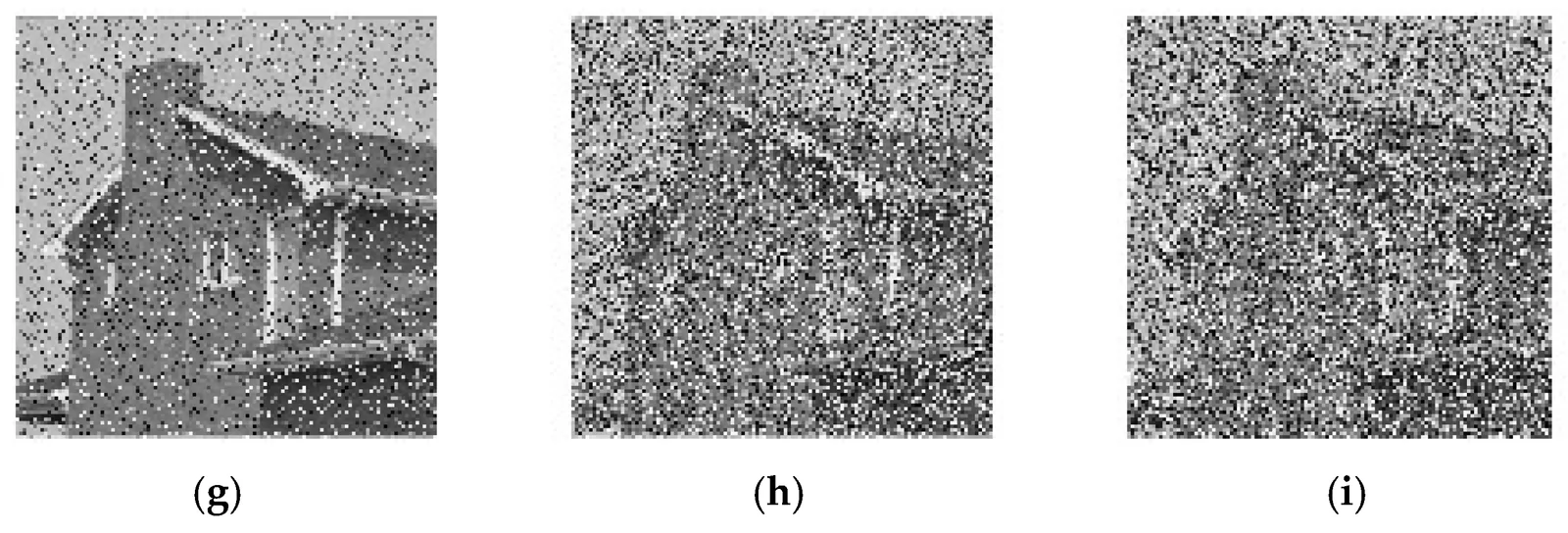
Decrypted images with Salt-and-Pepper noise of different intensities: (**a**,**d**,**g**) intensity is 0.015; (**b**,**e**,**h**) intensity is 0.04; (**c**,**f**,**i**) intensity is 0.06.

Under the interference of Salt-and-Pepper noise of different intensities, the identifiable valid information in the image gradually decreases. Figure 10 presents the experimental results, indicating that certain valid information can still be recognized at a noise intensity of 0.04. This further verifies that the proposed encryption scheme possesses a favorable capability to resist external noise interference.

#### 4.7.3. Structural Similarity Index (SSIM)

As a widely used metric, the structural similarity index (SSIM) evaluates the quality of decrypted images by measuring their similarity to the original ones. SSIM values range from −1 to 1, and values closer to 1 correspond to better restoration results.

(15) $$\mathrm{SSIM}(I,D)=\frac{\left(2\mu_{I}\mu_{D}+C_{1})(2\sigma_{ID}+C_{2}\right)}{\left(\mu_{I}^{2}+\mu_{D}^{2}+C_{1})(\sigma_{I}^{2}+\sigma_{D}^{2}+C_{2}\right)}$$

where $\mu_{I}$, $\mu_{D}$, $\sigma_{I}$ and $\sigma_{D}$ are the mean values and the variance values of I and D, respectively. $\sigma_{ID}$ is the covariance value between I and D. $C_{1}$ and $C_{2}$ are two regularization constants. The SSIM values are presented in Table 8. It can be concluded that the SSIM gradually degrades with the increase in noise intensity.

#### 4.7.4. Peak Signal-to-Noise Ratio

Similar to SSIM, the peak signal-to-noise ratio (PSNR) can also reflect the error level between two images. Larger PSNR values indicate greater similarity between the decrypted image and the original one.

(16) $$\mathrm{MSE}=\frac{1}{M\times N}\sum_{x=1}^{M}\sum_{y=1}^{N}{\left[O(x,y)-R(x,y)\right]}^{2}$$

(17) $$\mathrm{PSNR}=10\mathrm{lg}\frac{255\times 255}{\mathrm{MSE}}$$

where O(x, y) and R(x, y) are the pixel values of the original image and the decrypted one, respectively. The gray level is quantized with 8-bit binary, so n is 8. The proposed algorithm belongs to a lossless encryption algorithm, so its MSE value is 0. Table 8 displays the PSNR and MSE values of the images obtained after decryption when noise is added to the encrypted images. It is obvious that decryption quality gradually deteriorates with the increase in noise intensity.

### 4.8. Chi-Square Test

The randomness of encrypted images is typically evaluated using the chi-square test based on their statistical characteristics. Ideally, the pixel gray values of the cipher image are expected to be distributed uniformly without obvious fluctuations. If the chi-square result is lower than the threshold of 293.2478, the encryption performance is regarded as satisfactory.

(18) $$\chi^{2}=\sum \frac{(P_{i}-E_{i}{)}^{2}}{E_{i}}$$

where $P_{i}$ represents the occurrence frequency of different gray levels, $E_{i}$ is the ideal frequency. For a 256 × 256 image with intensity levels between 0 and 255, the expected frequency $E_{i}$ is 256. Table 9 shows that all test results meet the chi-square criterion.

### 4.9. Encryption Quality Analysis

Typically, irregular deviation (ID), uniform histogram deviation (UHD), and maximum deviation (MD) are used to evaluate image encryption quality.

#### 4.9.1. Irregular Deviations

ID is employed to evaluate image encryption quality

(19) $$\mathrm{ID}=\sum_{i=0}^{255}\left|G_{i}-G^{1}\right|$$

where $G_{i}$ denotes the absolute difference in the occurrence count of gray level i between the original and encrypted images, and $G^{1}=\sum_{i=0}^{255}G_{i}/256$. As listed in Table 10, a larger ID value implies that the original image content is significantly disturbed after encryption. This makes the ciphertext image harder to identify and analyze.

#### 4.9.2. Uniform Histogram Deviation

UHD [15] can also be used to evaluate image encryption quality.

(20) $$\mathrm{UHD}=\frac{\sum_{i=0}^{255}\left|I-I_{i}\right|}{M\times N}$$

where *i* denotes different gray levels, *I_i_* is the pixel count corresponding to gray level *i* in the encrypted image, I stands for the ideal uniform histogram of the image, while *M* and *N* represent the image height and width, respectively. Table 10 shows that the UHD values of all encrypted images approach 0. This indicates good encryption performance and negligible information leakage.

#### 4.9.3. Maximum Deviation

MD denotes the maximum gray-level difference between pixel pairs in the original image and its encrypted image.

(21) $$\mathrm{MD}=\max\left|P\left(i,\;j\right)\;-\;D\left(i,\;j\right)\right|$$

where *P*(*i*, *j*) and *D*(*i*, *j*) separately represent pixel values for the plaintext image and ciphertext image.

Table 10 lists the MD values of different encrypted images. The obvious discrepancies among these results demonstrate desirable encryption effectiveness.

### 4.10. Chosen-Plaintext Attack

**Figure 11:**
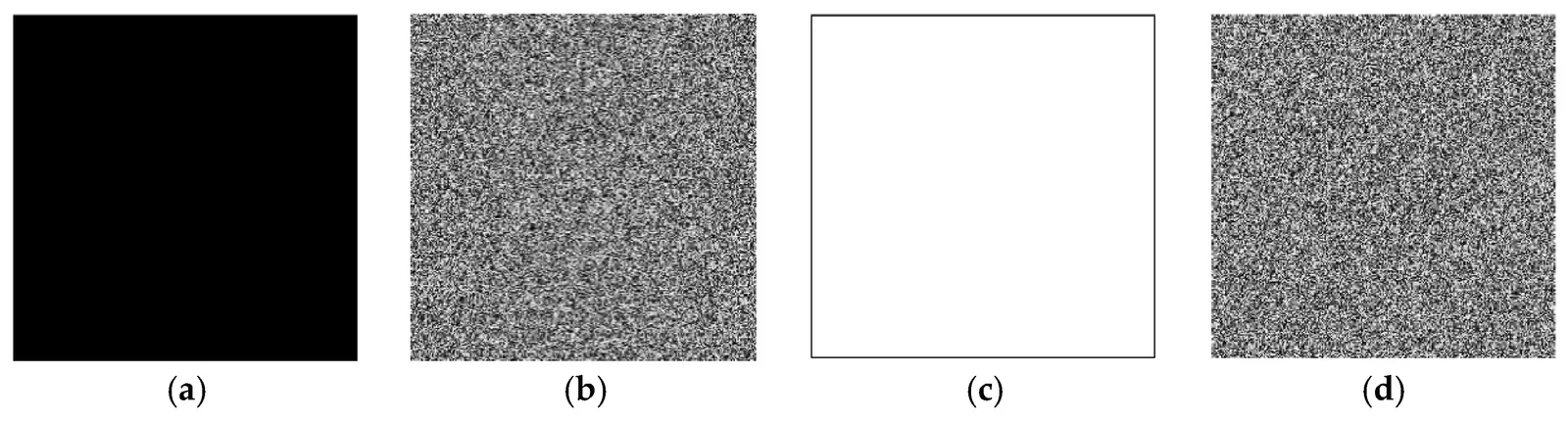
Experimental results: (**a**) pure black image; (**b**) encrypted pure black image; (**c**) pure white image; (**d**) encrypted pure white image.

The chosen-plaintext attack is a common attack model in image encryption. Attackers infer valid information by selecting specific plaintexts and acquiring the corresponding ciphertexts. Although FWA exhibits nonlinear characteristics, attackers often adopt pure white or pure black images to deduce the secret key. Figure 11 presents the encryption results of pure black and pure white images, from which no meaningful information can be visually distinguished. Consequently, the proposed encryption algorithm is capable of defending against the chosen-plaintext attack effectively.

### 4.11. Time Analysis

To evaluate the computational efficiency of the proposed encryption scheme, we quantitatively analyze the time consumption of different encryption stages, including the key-generation stage, reversible convolution diffusion stage, and the overall encryption process. The corresponding results are presented in Table 11, which further verify that the proposed algorithm achieves a good balance between encryption security and computational efficiency and is suitable for practical image-encryption applications.

## 5. Conclusions

An image encryption scheme that combines FWA with convolution operations is proposed. In this scheme, the 1D Logistic map is used to regulate FWA in generating new pseudo-random sequences. This method enhances the security of 1D chaotic maps and avoids the high computational cost of high-dimensional chaotic systems in practical applications. Moreover, a reversible convolution operation is designed. Pixel values are correlated with both the pseudo-random sequences generated by FWA and the pixels preselected prior to convolution. In addition, convolution kernels and selected columns are not reused in multiple convolution rounds, thereby further enhancing the algorithm’s sensitivity and its robustness against various attacks. Experimental results demonstrate that the integration of chaotic systems with FWA achieves a large key space, high key sensitivity, and strong resistance to brute-force and statistical attacks. Quantitative tests show that the information entropy of the encrypted image is higher than 7.988, while the NPCR and UACI values exceed 99.9% and 33.36%, respectively. These indicators further verify the security and effectiveness of the proposed scheme. Hence, the proposed scheme achieves favorable security and outstanding encryption performance. In future work, higher-dimensional chaotic systems can be introduced to investigate multi-image encryption with a proper trade-off between efficiency and computational complexity.

## Figures and Tables

**Table 1:** NIST test results.

Test Name	*p*-Value (*p* ≥ 0.01)	Result (√/×)
Frequency	0.8137	√
Block frequency	0.0326	√
Runs	0.0509	√
Longest run of ones in a block	0.1972	√
Binary matrix rank	0.6281	√
Discrete Fourier transform	0.9207	√
Non-overlapping template matching	0.7449	√
Overlapping template matching	0.6338	√
Maurer	0.3143	√
Random excursions variant	0.1827	√
Linear complexity	0.5993	√
Serial	0.2805	√
Approximate entropy	0.7419	√
Cumulative sums	0.9491	√
Random excursions	0.3103	√

**Table 2:** Example of convolution calculation in the literature [35].

	Lh	Input Matrix	Convolution Kernel	Output Matrix	Normalized Output Matrix
1	2	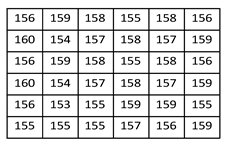	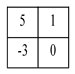	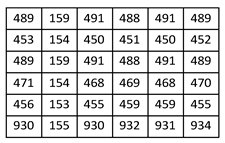	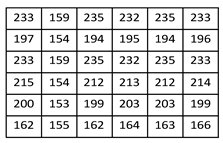
2	4	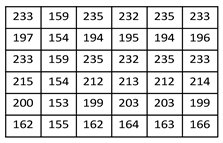	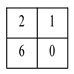	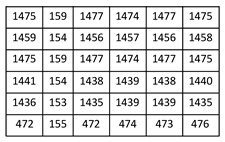	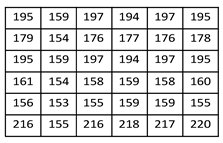
3	2	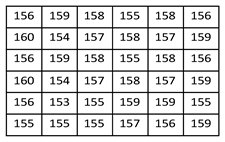	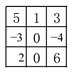	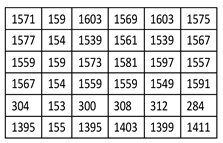	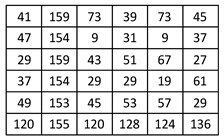
4	4	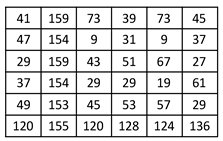	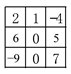	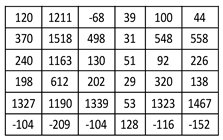	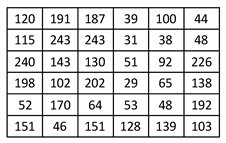

**Table 3:** Example of the convolution calculation as proposed in this paper.

	Lh	Input Matrix	M1	Convolution Kernel	Output Matrix	Normalized Output Matrix
1	2	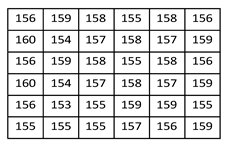	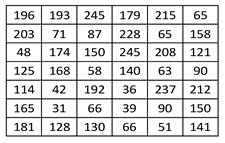	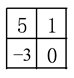	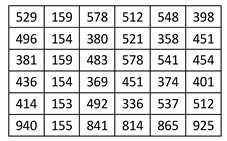	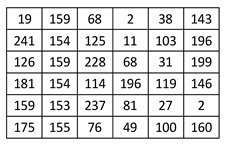
2	4	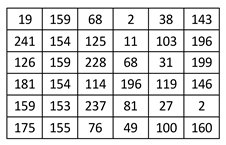	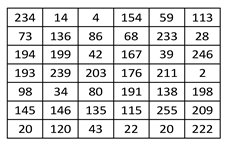	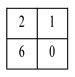	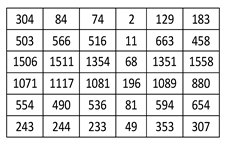	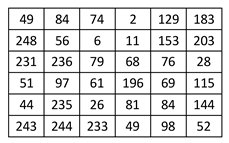
3	2	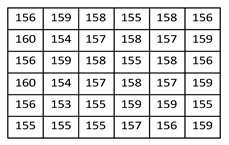	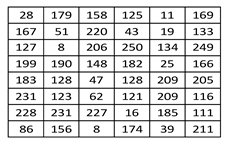	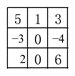	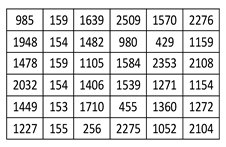	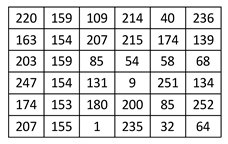
4	4	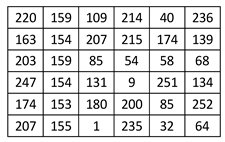	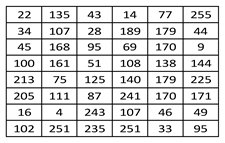	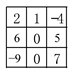	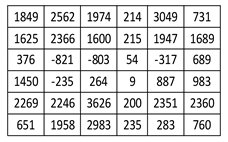	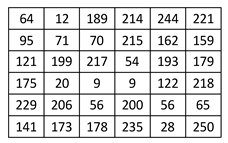

**Table 4:** Deconvolution operations.

	Lh	Input Matrix	M1	Convolution Kernel	Output Matrix	Normalized Output Matrix
1	4	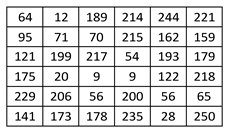	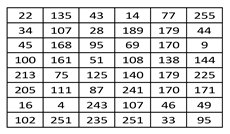	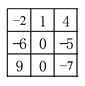	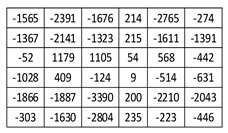	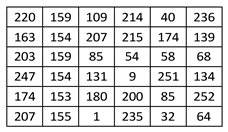
2	2	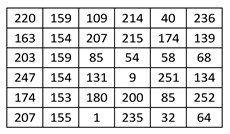	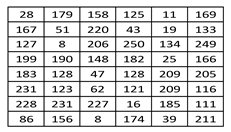	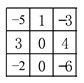	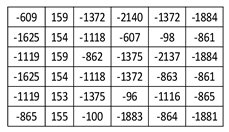	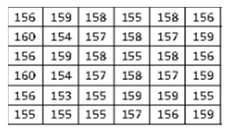

**Table 5:** Information entropy values for test images (bits).

Algorithm	Images	Plain Image	Encrypted Image
Proposed algorithm	Cameraman	7.0097	7.9903
Proposed algorithm	Peppers	6.8609	7.9893
Proposed algorithm	Clock	6.7057	7.9889
Proposed algorithm	Female	7.2575	7.9888
Proposed algorithm	house	6.4961	7.9896
Hu et al. [39]	Cameraman	7.0097	7.9831
Wu et al. [40]	Cameraman		7.9935
Sang et al. [41]	Cameraman		7.9661

**Table 6:** Correlation coefficients for each test image in different directions.

Algorithm	Images	State of Affairs	Horizontal	Vertical	Diagonal
Proposed algorithm	Cameraman	Plain image	0.9289	0.9550	0.8932
		Encrypted image	0.0070	0.0014	−0.0011
Proposed algorithm	Peppers	Plain image	0.9477	0.9472	0.9314
		Encrypted image	0.0051	0.0329	−0.0226
Proposed algorithm	Clock	Plain image	0.9497	0.9727	0.9265
		Encrypted image	0.0084	0.0104	0.0029
Proposed algorithm	Female	Plain image	0.9427	0.9742	0.9272
		Encrypted image	−0.0401	0.0127	0.0173
Proposed algorithm	House	Plain image	0.9727	0.9497	0.9306
		Encrypted image	−0.0207	0.0202	0.0191
Hu et al. [39]	Cameraman	Encrypted image	−0.0166	−0.0027	0.0043
Wu et al. [40]	Cameraman	Encrypted image	−0.0036	0.0048	0.0073
Sang et al. [41]	Cameraman	Encrypted image	−0.0280	0.0385	−0.0092

**Table 7:** NPCR and UACI of different test images.

Algorithm	Image	NPCR (%)	UACI (%)
Proposed algorithm	Cameraman	99.5917	33.3621
Proposed algorithm	Peppers	99.6034	33.4010
Proposed algorithm	Clock	99.6396	33.3693
Proposed algorithm	Female	99.6178	33.3957
Proposed algorithm	House	99.6021	33.3724
Maniyath et al. [38]	Peppers	99.6231	33.2749
Aziz et al. [47]	Peppers	99.5650	30.9131
Dong et al. [48]	Peppers	99.5800	33.0200

**Table 8:** SSIM, MSE, and PSNR of test images at different intensities of salt-and-pepper noise.

	Intensities		0.015	0.04	0.06
Images					
Cameraman		MSE	1984	3957	5862
		PSNR (dB)	35.2570	31.5060	29.7800
		SSIM	0.2458	0.1123	0.0398
Peppers		MSE	2001	4908	5505
		PSNR (dB)	35.5105	31.0498	30.3038
		SSIM	0.2052	0.0801	0.0687
Clock		MSE	2128	4892	7170
		PSNR (dB)	33.7874	29.1984	28.1774
		SSIM	0.2073	0.0874	0.0605
Female		MSE	1844	3855	6407
		PSNR (dB)	35.0509	30.9953	29.4091
		SSIM	0.1692	0.0713	0.0273
House		MSE	1787	4416	4963
		PSNR (dB)	33.4548	29.1947	28.6239
		SSIM	0.1591	0.0539	0.0479

**Table 9:** $\chi^{2}$ of different images.

Image	Original Image	Encrypted Image	Result
Cameraman	110,973.3047	222.3438	Pass
Female	68,973.1016	250.5156	Pass
House	300,852.0781	236.6797	Pass
Peppers	35,378.2500	260.9688	Pass

**Table 10:** Encryption quality detection indicators for different images.

Image	ID	UHD	MD
Cameraman	32,347.5313	0.0472	248
Peppers	23,909.7188	0.0502	219
Clock	47,778.2188	0.0562	245
Female	25,947.9375	0.0510	250
House	48,779.6875	0.0471	234

**Table 11:** Encryption and decryption times at different stages.

Encryption Time for Different Stages (s)		Decryption Time for Different Stages (s)	
Key-generation	0.2749	Key-generation	0.1913
Convolution	0.1640	Inv-convolution	0.1521
Encryption process	0.4908	Decryption process	0.3760

## Data Availability

The data presented in this study are available upon request from the corresponding author.
